# Cultural dimensions in online purchase behavior: Evidence from a cross-cultural study

**DOI:** 10.1007/s43039-021-00022-z

**Published:** 2021-04-07

**Authors:** Francesca Pratesi, Lala Hu, Riccardo Rialti, Lamberto Zollo, Monica Faraoni

**Affiliations:** 1 Project and Finance Consulting, Resolvo S.R.L., Florence, Italy; 2 Department of Economic and Business Management Sciences, Catholic University of the Sacred Heart, Milan, Italy; 3 Department of Economics and Management, University of Florence, Florence, Italy

**Keywords:** Consumer culture, E-commerce, Online purchase behavior, Perceived risk, Trust, Website usability

## Abstract

The objective of this research is to investigate how cultural differences affect consumers’ online purchase behavior. We reviewed the recent literature on cross-cultural studies on online behavior and building on Hofstede’s theory of cultural dimensions and the theory of planned behavior (TPB), we developed a conceptual model exploring how the dimensions of national culture influence perceptions of website usability, trust, and perceived risk, which in turn impact on intention to use and online purchase behavior. A web-based questionnaire was distributed to a sample of 350 European and Asian consumers actively using Alibaba e-commerce platforms. The conceptual model was validated through a confirmatory factor analysis (CFA), while structural equation modelling (SEM) was used to empirically test the hypothesized relationships among variables. Results showed how culture significantly influenced website usability and perceived risk in European consumers and, in turn, their intention and behavior. Differently, culture significantly influenced trust of Asian consumers, as well as their intention and online behavior. With this study, we contribute to the literature on consumer online purchase behavior from a cross-cultural perspective. As culture emerged among the significant antecedents of mechanisms explaining online purchase behavior, e-tailers should tailor digital marketing strategies according to consumer cultural differences.

## Introduction

Online shopping is a global marketing megatrend (Von Briel, 2018), making an unlimited range of products and services available for consumers all over the world (Pappas et al., 2016; Quelch & Klein, 1996). Such a phenomenon has greatly been exacerbated by the COVID-19 pandemic, which forced home the most of consumers permitting them online purchases only (Sheth, 2020). The leading global e-retailers are Alibaba and Amazon (Statista, 2020), which were founded in two different countries, China and the United States, respectively. The two companies have also undergone two different internationalization processes. While Amazon was established in 14 international markets as of 2017, Alibaba operates at the international level through Aliexpress, a marketplace to buy products and services directly from sellers in China present in Europe, Russia, and America, and Lazada, an e-commerce company acquired in 2016 mainly present in other Asian countries (Wu & Gereffi, 2018).

Recent studies determined how consumer perceptions of the trustworthiness of e-commerce platforms might differ: for example, Western consumers are more skeptical toward China-based Alibaba than US-based Amazon or Italy-based Yoox (Kwak et al., 2019). Moreover, there is a higher perceived risk of purchasing counterfeit products on Alibaba, in particular, among Western consumers as opposed to Eastern ones (Valarezo et al., 2018). Scholars have pointed out that one of the main reasons underlying this phenomenon is the different cultural backgrounds of consumers, as the latter dramatically shapes consumer perceptions (Marino et al., 2020; Peng et al., 2017). Indeed, elements such as national pride or individuals’ status in their societies, for example, may drive consumers’ preferences (Smith et al., 2013). Similarly, extant research suggested that customer final purchase decisions are influenced by perceptions of ease of use and risk involved in these platforms (Faraoni et al., 2019). E-commerce platforms characterized by safe payment procedures and easily understandable by consumers have indeed a competitive advantage over competitors (Zollo et al., 2020).

Accordingly, both the literature on e-commerce and the one on cultural differences are still growing in response to research quests coming from the practitioners’ world. However, some research gaps still exist. Indeed, limited research has explored how culture is related to different customer evaluations concerning website usability, trust, and perceived risk. On the one hand, the cultural dimension theory has been used at most by international marketing scholars to evaluate the entrance in new markets through physical shops and to develop innovative communication strategies (Litvin, 2019). On the other hand, e-commerce research mostly focused on the exploration of consumers’ behavioral dynamics in western markets, which are also the most mature ones (Rehman et al., 2020).

To cope with these gaps, we conducted an empirical study adopting a cross-cultural perspective. In detail, we investigated how cultural elements influence attitudes toward the use of a specific online platform and customer online purchase intentions. A conceptual model based on Hofstede’s (2001) cultural dimensions theory and Ajzen’s (1991) theory of planned behavior (TPB) was developed. As the focus of this research concerns the observation of cultural difference’s importance among Alibaba customers, a cross-country data collection was presented, involving respondents in Europe and Asia. Such an approach allowed us to perform a direct comparison between two different group of consumers. Therefore, we contribute to the existing literature on cross-cultural research on online shopping behavior by analyzing the cultural effects of the use of an international e-commerce platform, and if there are any differences in the online purchasing behavior of consumers from different backgrounds. Specifically, we will shed some lights on the importance of cultural differences on perceived website usability, trust, and risk.

The remainder of this paper is structured as follows: we provide a literature review on cultural dimensions and online purchase behavior, then we present the hypotheses and variables of the proposed model. The following section discusses the results of the structural equation model, which show that culture influences the trust of Asian customers more than the trust of European customers. From that, implications for theory and practice are developed.

## Literature review

The most widely adopted framework to analyze cultural differences among marketing and management scholars is Hofstede’s (2011) cultural values model, originally developed based upon employees of IBM and found by subsequent research to well represent broad differences in national culture (Smith et al., 2013). Hofstede (1991) identified five universal dimensions of culture (power distance, individualism, masculinity, uncertainty avoidance, and long-term orientation), which are defined as the following:

- *Power distance* refers to social inequality, including the relationship of the individual with authority. High power distance nations include India, China, and Mexico, whereas low power distance nations mainly include Western countries, as in the case of the United States, Australia, and Israel (Rinne et al., 2012).
- *Individualism‐collectivism* represents the relationship between the individual and the group. Under this dimension, adherence to the norm and group decisions is more important in Asian countries than individualistic needs or wants (Fam & Waller, 2003).
- *Masculinity‐femininity* concerns the social implications of having been born as a boy or a girl. Except for Japan, most East Asian countries are ranked lower on masculinity than most of the Western countries (Hofstede et al., 1998).
- *Uncertainty avoidance* refers to ways of dealing with uncertainty, relating to the control of aggression and the expression of emotions. While East Asian countries score quite similarly on other dimensions, uncertainty avoidance has been suggested as one of the most defining cultural differences among East Asians. For example, the uncertainty avoidance index shows that Japan and Taiwan score high on uncertainty avoidance while China and Singapore score low (Hofstede et al., 2010).
- *Long-term orientation* focuses on the degree to which people concentrate their efforts on the future rather than the present and the past (Minkov & Hofstede, 2011). The four highest-scoring countries on this dimension are all East-Asian, that are South Korea, Taiwan, Japan, and China (Hofstede & Minkov, 2010).
  Additionally, in more recent times, literature focused on cultural difference tried to expand the number of dimensions to be considered (Minkov & Hofstede, 2011). In this perspective, an additional dimension has been considered among the ones characterizing a national culture:
- *Indulgence-restraint*, which refers to the level of self-gratification that a society allow the individual to obtain with his/her own means. Usually, indulgence shows higher level in western countries and Oceania, while national cultures from Asian, African and South America show higher level of restraint. This notwithstanding, such a dimension had been scarcely used by prevailing literature, and researches including it are mostly in seminal phases (Sun et al., 2019).

From the cross-cultural point of view, one main area of investigation in online behavior is represented by trust, which is defined as a group of beliefs held by a person derived from his or her perceptions about certain attributes (Flavián et al., 2006). Trust represents a social antecedent of online shopping adoption, therefore e-tailers should build their websites in order to communicate trust to users (Gefen et al., 2003). According to Chen and Dibb (2010), key dimensions that affect website trust include usability, security and privacy assurance, and product information quality. From a cross-cultural point of view, Hallikainen and Laukkanen (2018) found that long-term orientation and collectivism interrelate with disposition to trust in e-commerce. Trust is found to be higher in collectivist cultures as compared to individualist cultures, confirming previous research (Doney et al., 1988; Huff & Kelley, 2003). Regarding long-term orientation, it has a positive effect on disposition to trust suggesting that in long-term orientated cultures, business relationships are typically built on a long-lasting basis (Hallikainen & Laukkanen, 2018).

Previous studies have also analyzed the impact of cultural differences on ease of use, which represents the degree to which a person believes that using a particular technology would be free of effort (Davis, 1989). In the case of Internet navigation, “ease of use” is referred to as “usability,” a term that is more frequently adopted in e-commerce literature (Flavián et al., 2006). In a more recent study, Smith et al. (2013) found a direct relationship between perceived usability and behavior intention among American users. Belanche et al. (2012) conducted a study with a Spanish sample. Their results showed that website usability affects satisfaction, which in turn positively affects consumer intention to use a website.

Another area of investigation is represented by perceived risk, which is defined as the potential for loss in pursuing a desired outcome during the process of online shopping (Ko et al., 2004). Perceived risk may reduce consumer perceptions of control, which can influence their behavior with e-tailers (Park et al., 2012). It has also been found to act as moderator in the relationship between perceived website usability and consumer satisfaction (Belanche et al., 2012). Therefore, companies should ensure website usability as it helps overcome those consumers that perceive a high risk in using a website.

From the cultural point of view, most studies on e-commerce behavior have analyzed U.S.-based samples (Cayla & Arnould, 2008). A few studies have adopted a cross-cultural perspective; for example, Ko et al. (2004) compared the differences in perceived risk between Koreans and Americans, suggesting that Korean users, who belong to a collectivist country, display a higher level of social risk. Capece et al. (2013) made a cross-cultural study involving Italian and Chinese consumers. The main dimensions influencing trust and intention to use e-commerce in Italy are represented by power distance and individualism, while long-term orientation and uncertainty avoidance are the cultural values that influence Chinese consumers’ acceptance of online shopping. Peña-García et al. (2020) analyzed Spanish and Columbian consumers finding that buying impulse is an antecedent of online purchase intention in markets with a short-term orientation such as Columbia, therefore appropriate design strategies for online stores that can trigger purchase should be developed.

In spite of this growing body of literature, to our best knowledge little research has considered -following a holistic approach- the simultaneous impact of cultural dimensions on intention to use an online e-commerce platform, privacy concerns and trust. Such a situation is counterintuitive as cultural dimensions contribute significantly to shape consumer behavior. According to De Mooij and Hofstede (2011), indeed, different cultures may cause differences in cognitive processes, information seeking and processing, and adoption of innovation. Similarly, they may cause dissimilarities in emotion arousal and inner motivations of consumers (Zollo et al., 2018). As a consequence, to better investigate and unfold these mechanisms, the present research will try to answer the following research question:

### RQ

What is the impact of cultural dimensions on consumer’s website usability, perceived risk and trust; and, in turn, how do these affect their intention to use an online e-commerce platform?

## Hypotheses development

The aim of this research is to evaluate how consumer cultural values—which derive from their individual cultural backgrounds—influence their perceptions concerning an e-commerce platform and their subsequent intentions to use it to purchase a product. In order to do so, a structural model was jointly developed by the four authors. The structural model is based on the intertwining of Hofstede’s cultural dimensions’ theory (1991) with Ajzen’s (1991) theory of planned behavior (TPB).

### The impact of consumer cultural values on their perceptions concerning an e-commerce platform

Hofstede’s (1991) cultural dimensions’ theory argues that individual behavior is shaped by five dimensions depicting any national or regional culture. As previously assessed, European and Asian consumers are characterized by different cultural values with respect to their perceptions of power distance, individualism/collectivism, uncertainty avoidance, masculinity/femininity, and long-term/short-term orientation. National cultures shape the behavior of consumers in an online environment (Capece et al., 2013) leading to different perceptions of the e-commerce platform usability, trust, and involved risk.

In general, Asian countries are characterized by a higher uncertainty avoidance and long-term orientation than Western countries, making them less prone to accept risks. As a consequence, perceived usability of an e-commerce platform will be lower in Asian consumers. This could influence the willingness to adopt and use a new technology (Baptista & Oliveira, 2015). Hence, Asian customers might be more reluctant to engage in online shopping since e-commerce is perceived as having uncertain outcomes (Yoon, 2009). Therefore, we propose:

#### H1

Culture affects consumer perceptions of e-commerce platform usability. European consumers (H1a) may perceive an e-commerce platform as easier to use than their Asian counterparts (H1b).

Similarly, Asian consumers may perceive a higher risk in using a not-fully-known platform. The level of trust will thus be affected; indeed, someone wishing to avoid an uncertain outcome will probably be less prone to trust something new (Hwang & Lee, 2012). Customers from high power distance countries, like Asian countries, tend to have less trust toward an online shopping mall than do customers from low power distance countries (Yoon, 2009). Concerning individualism/collectivism dichotomy, individualist societies such as Europeans countries, are more prone to question authority, and, consequently, they are less afraid of switching to a different e-commerce platform (Hofstede, 2011). The opposite is true for Asian collectivistic societies, which are integrated into deeply structured familiar or social networks. Therefore, we propose:

#### H2

Culture affects consumer trust in an e-commerce platform. European consumers may be more prone to trust a platform (H2a) than their Asian counterparts (H2b).

A similar effect is caused by the orientation toward masculinity in respect to femininity (Hofstede et al., 1998). In many countries, both men and women share a common need for achievement, assertiveness, and success. Such countries, as is the case with European ones, are characterized by a diffused sense of masculinity. In Asian countries that are characterized by femininity, it has been observed that both men and women share a common orientation toward modesty. Yet in these countries it is also true that women are frequently viewed as more submissive and less interested in career advancements. The gender gap is therefore wider in feminine cultures (Hofstede, 1991). Such a trait of national culture could noticeably influence the intention to use an e-commerce platform. European consumers (both men and women) may be more prone to accept a risk, and therefore to use technology (Stafford et al., 2004; Venkatesh & Morris, 2000), while Asian ones tend to be more concerned about risk. Similarly, the more confident European consumers may perceive a platform as easier to use and may be more prone to trust the e-tailer. Based on these assumptions, we propose:

#### H3

Culture affects consumer perceptions of risk related to the use of a new e-commerce platform. Asian consumers may perceive risks as greater (H3a) than their European counterparts (H3b).

### Consumer perceptions and intention to use an e-commerce platform

According to theory of planned behavior (TPB), perceptions or beliefs are the principal drivers of consumer intentions. This phenomenon has also been explored in regard to consumer intentions to use a digital technology like e-commerce platforms (Bhattacherjee, 2000).

Among the main factors influencing consumer intentions to use an e-commerce platform, perceived usability plays a pivotal role. Flavián et al. (2006), for example, identified perceived usability as a relevant antecedent to consumer willingness to adopt an e-commerce platform. Indeed, perceived usability could influence consumer satisfaction, trust, and loyalty, thus improving consumer intention to use a platform (Chen & Dibb, 2010). Consumer perceptions concerning their capability to use a technology could also influence the perceived utility they attribute to it. Similarly, the perceived degree of usability will influence their relationship with e-commerce platforms (Biraghi et al., 2020). Consumers, in fact, tend to develop better relationships with platforms that they are capable of using completely (Zhang et al., 2011). Cultural differences also play a role in this regard. More confident consumers, such as European ones, may more quickly perceive a technology as more usable. Thus, it is plausible that the relationship between perceived usability and intention to use will be generically stronger in European consumers. Therefore, we propose:

#### H4

Website usability positively influences consumer intention to use an e-commerce platform, but the relationship between website usability and intention to use will be stronger in European consumers (H4a) than Asian ones (H4b).

Consumers develop trust whether the information provided by the vendor are unbiased, clear, and verifiable (Rialti et al., 2018). The existence of trust is fundamental; indeed, trust is the base of any marketing strategy wishing to build a relationship with consumers. Trust toward the e-commerce platform is another relevant antecedent of intention to use an e-commerce platform. According to Kim and Peterson (2017), consumers tend to make more use of platforms that ensure safe payments, sell not-counterfeited products, and warrant that products come from certified vendors. Such a phenomenon is related to the cultural background of consumers and country-of-origin of the e-commerce platform. European consumers do not tend to trust Chinese e-commerce platforms; thus, in the specific Alibaba case (or in analogous cases), the relationship between trust and intention to use the platform will be stronger in Asian consumers. Therefore, we propose:

#### H5

Trust positively influences both European and Asian consumer intentions to use an e-commerce platform; but the relationship between trust and intention to use will be stronger in Asian consumers (H5b) than European ones (H5a);

In regard to perceived risk, Pavlou (2002) identified perceived risk as one of the main factors hampering the success of e-commerce platforms. Consumers negatively perceive platforms characterized by weak data protection policies or populated by scammers. Accordingly, high perceived risk influences the use of an e-commerce platform as consumers do not wish to have their privacy and data stolen. In regard of this matter, both European and Asian consumers are concerned about perceived risk, as private information are relevant to both of them (Kim & Peterson, 2017). Therefore, we propose:

#### H6a, b

Perceived risk negatively affects both European and Asian consumer intentions to use an e-commerce platform.

### Intention to use an e-commerce platform and online purchase behavior

Marketing research has highlighted how consumer online purchase behavior is a frequent outcome of intention to use an e-commerce platform. Such a phenomenon is coherent with Ajzen’s (1991) TPB. Indeed, it has been observed that consumers willing to use an online platform will probably also try to complete a transaction online (Goldsmith, 2002). This assumption has proven to be true in any context worldwide (Tong, 2010). Therefore, we propose:

#### H7a, b

Intention to use an e-commerce platform positively influences the online purchase behavior of both European and Asian consumers.

### Proposed conceptual model

**Fig. 1:**
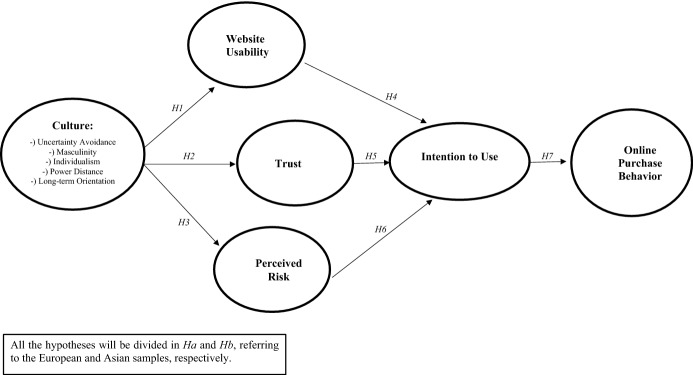
Conceptual model

Building on the seven hypotheses, the authors developed the following conceptual model (Fig. 1). Structural equation modeling was selected as the most suitable methodology to test it empirically.

## Methods

### Data collection and sample

A cross-sectional study was conducted from September to December 2018 using the Prolific platform to collect representative samples of European and Asian consumers actively using the Alibaba e-commerce channel (Rialti et al., 2017). Prolific allows the avoidance of typical biases of the survey-based approach, such as social desirability bias (Podsakoff et al., 2003) and non-response bias (Rogelberg & Stanton, 2007). Alibaba was chosen as the focus of the analysis as it is a global player in the e-commerce sector that started its internationalization expansion since 2009 (Anwar, 2017). Operating among multiple markets, it allows a cross-cultural analysis to explore the characteristics of the online behavior of its customers based in different countries. A twelve-page questionnaire in English incorporating 51 items of validated scales and five control variables was developed and pre-tested by a student panel of 10 respondents enrolled in an Italian university marketing course. English has been selected as the main language of research due to the necessity to create a leveled playing field, using a neutral language for all the respondents. Next, three Italian professors with expertise on consumer behavior completed the questionnaire to check its clarity and ease of completion (Zollo et al., 2017). After these pre-tests, no changes were made to the final questionnaire which was then completed by 350 respondents, 175 from Europe and 175 from Asia. This sample size is considered as adequate in line with the SEM pertinent literature (see Hair et al., 1998).

The European sample was mainly composed of female respondents (67.2%) as in the Asian sample (58%). In both European and Asian samples, Millennials (those born between 1980 and 2000; Goldman Sachs, 2021) represented the majority of the respondents (34.5% and 45.4%, respectively). Consistently with recent studies (Zollo et al., 2020), Millennials are the generation of “techno-savvy” digital natives characterized by a high level of experience concerning e-commerce and social media. Hence, they represent an adequate sample for the purpose of our research (Bonera et al., 2020). Concerning occupation, students represented most of the sample (Europe = 49.4%, Asia = 46.0%) followed by white-collar workers (29.9% and 22.4%, respectively), and blue-collar workers (11.5% and 9.2%, respectively). Finally, the UK represented the largest country of origin (37.6%) in the European sample, whereas China was the largest country of origin (21.9%) in the Asian sample.

### Measures

All the items on the questionnaire were measured on a 7-point type Likert scale, from strongly disagree (1) to strongly agree (7). For *website usability*, the seven-item scale by Flavián et al. (2006) was used (e.g., “The structure and contents of this website are easy to understand”). For *trust*, the sixteen-item instrument by Teo and Liu (2007) was used (e.g., “This e-commerce vendor provides reliable information”). *Perceived risk* was captured through Teo and Liu’s (2007) four-item scale (e.g., “There is a high probability of losing a great deal by purchasing online from this e-commerce vendor”). The three-item instrument by Yoon (2009) was used to capture *intention to use* (e.g., “I am very likely to provide the online shopping mall with the information it needs to better serve my needs”). We used the six-item scale by Teo and Liu (2007) for *online purchase behavior* (e.g., “The likelihood that I would return to this vendor’s website is very high”). Finally, the multi-dimensional construct *culture* was captured using the fifteen-item instrument validated by Yoon (2009) which is composed of five sub-dimensions: *power distance* (e.g., “Managers should make most decisions by themselves”), *individualism* (e.g., “Individual rewards are more important than group welfare”), *masculinity* (e.g., “A job with high earnings is better than a job with quality of life”), *uncertainty avoidance* (e.g., “When starting a new job, I fear doing it”), and *long-term orientation* (e.g., “I consider myself as a persistent worker”).

### Reliability and correlation analysis

Preliminary statistical analyses were conducted using SPSS (v.25) (Field, 2013). As shown in Tables 1 and 2, all Cronbach’s alpha values were higher than 0.7 in both samples as required (Hair et al., 2006). Thus, all the scales were statistically reliable and were retained in the analysis.

**Table 1:** European sample: Correlation matrix and scales’ reliabilities

Variable	Mean	SD	1	2	3	4	5	6	7	8	9	10
1. Website Usability	4.69	0.98	*(0.95)*									
2. Trust	5.53	1.00	0.58*	*(0.88)*								
3. Perceived Risk	3.11	1.05	−0.20*	−0.23*	*(0.92)*							
4. Intention to Use	4.28	1.11	0.42*	0.59*	−0.19*	*(0.83)*						
5. Online Purchase Behavior	5.17	1.14	0.36*	0.57*	−0.26*	0.87*	*(0.96)*					
6. Power Distance	4.85	0.92	0.20*	0.34*	0.21*	0.43*	0.36*	*(0.80)*				
7. Individualism	5.19	1.06	0.18*	0.20*	0.21*	0.20*	0.14	0.50*	*(0.73)*			
8. Masculinity	5.25	1.12	0.20*	0.24*	0.26*	0.25*	0.23*	0.52*	0.62*	*(0.76)*		
9. Uncertainty Avoidance	2.80	1.05	0.08	0.05	0.17*	0.04	−0.11	−0.30	0.22*	0.11	*(0.73)*	
10. Long−term Orientation	3.50	0.92	0.05	0.10	0.18*	0.09	−0.05	−0.10	0.18*	0.01	0.15*	*(0.72)*

*Cronbach’s alpha values are reported on the diagonal*

*p-value < 0.01

**Table 2:** Asian sample: Correlation matrix and scales’ reliabilities

Variable	Mean	SD	1	2	3	4	5	6	7	8	9	10
1. Website Usability	4.20	1.12	*(0.94)*									
2. Trust	4.85	0.98	0.65*	*(0.86)*								
3. Perceived Risk	4.18	1.10	−0.22*	−0.31*	*(0.87)*							
4. Intention to Use	3.88	0.89	0.44*	0.65*	−0.27*	*(0.77)*						
5. Online Purchase Behavior	4.45	0.95	0.42*	0.62*	−0.34*	0.85*	*(0.96)*					
6. Power Distance	4.20	1.15	0.11	0.22*	0.21*	0.08	0.09	*(0.75)*				
7. Individualism	3.40	1.11	0.03	0.17*	0.27*	0.11	0.15	0.44*	*(0.82)*			
8. Masculinity	4.85	0.99	0.09	0.11	0.29*	−0.05	−0.02	0.51*	0.44*	*(0.74)*		
9. Uncertainty Avoidance	3.25	0.88	−0.04	0.05	0.21*	0.35*	0.33*	0.13	0.28*	0.18*	*(0.84)*	
10. Long-term Orientation	3.62	1.05	0.12	0.04	0.20*	0.10	−0.12	−0.15	0.15*	0.02	0.18*	*(0.74)*

*Cronbach’s alpha values are reported on the diagonal*

*p−value < 0.01

Interestingly, European respondents showed higher mean values of *individualism* (M = 5.19, SD = 1.06) than their Asian counterparts (M = 3.40, SD = 1.11) and higher mean values of *masculinity* (Europe: M = 5.25, SD = 1.12; Asia: M = 4.85, SD = 0.99). In contrast, the Asian respondents showed higher values of *uncertainty avoidance* (M = 3.25, SD = 0.88) than the European respondents (M = 2.80, SD = 1.05) and higher values of *perceived risk* (M = 4.18, SD = 1.10; M = 3.11, SD = 1.05, respectively).

Concerning the correlation analysis, the highest Pearson’s *r* values were between *intention to use* and *online purchase behavior* (Europe: *r* = 0.87; Asia: *r* = 0.85), consistent with the TPB (Ajzen, 1991). In both samples, *trust* resulted as one of the most correlated variables with *intention to use* (Europe: *r* = 0.59; Asia: *r* = 0.65) and with *online purchase behavior* (Europe: *r* = 0.57; Asia: *r* = 0.62). With respect to cultural dimensions, the European sample showed the highest correlation between *power distance* and *intention to use* (*r* = 0.43), while in the Asian sample, the variable *uncertainty avoidance* was highly correlated with *intention to use* (*r* = 35).

### Validity testing

We conducted a confirmatory factor analysis (CFA) to check the validity of the hypothesized model (Hair et al., 2006). AMOS (v.25) was used to assess the fit indexes of the considered constructs (Arbuckle, 2013): culture as a second-order construct comprising uncertainty avoidance, masculinity, individualism, and power distance; website usability, trust, and perceived risk as antecedents of intention to use; and online purchase behavior as the main outcome variable of the model (see Fig. 1).

First, the CFA of both the European and Asian samples showed that all the path coefficients between the items and the latent variables (the “factor loadings”) were statistically significant (p < 0.01) and higher than 0.5 as required (Hair et al., 2006). Specifically, all four sub-dimensions of culture showed adequate factor loadings. For Europe, *uncertainty avoidance* = 0.67; *masculinity* = 0.80; *individualism* = 0.76; *power distance* = 0.67; *long-term orientation* = 0.62. For Asia, *uncertainty avoidance* = 0.68; *masculinity* = 0.70; *individualism* = 0.65; *power distance* = 0.69; *long-term orientation* = 0.64. Hence, all the constructs were retained in the analysis. Next, we calculated the goodness-of-fit measures to verify the satisfactory parsimony and validity of the proposed six-factor model (Bagozzi & Yi, 1988). The first set of indicators referred to the absolute fit indexes. For the European sample, the relative Chi-square statistics (Chi-square divided per degrees of freedom) suggested a satisfactory fit of the model (χ^2^/df = 2.895) as it was lower than three (Bentler, 1990); the goodness-of-fit index (GFI = 0.948) also showed a good fit of the model as it was higher than 0.90 as required (Bagozzi & Yi, 1988); similarly, the root mean square error of approximation (RMSEA = 0.062) suggested an acceptable model fit as it was lower than 0.07 (Bentler, 1990). For the Asian sample, we found satisfactory values of absolute fit indexes: the relative Chi-square statistics (χ^2^/df = 2.765), the GFI (0.965), and the RMSEA (0.058) showed a good fit for the model (Bagozzi & Yi, 1988; Bentler, 1990).

The second set of indicators referred to the relative fit indexes. For the European sample, the comparative fit index (CFI = 0.952), the incremental fit index (IFI = 0.945), the normed fit index (NFI = 0.940), and the Tucker-Lewis index (TLI = 948) showed adequate levels higher than 0.90 as required (Bagozzi and Yi, 1998). Similarly, the relative fit indexes of the Asian sample were all higher than 0.90 (CFI = 0.975; IFI = 0.967; NFI = 0.960; TLI = 0.968).

Hence, the goodness-of-fit measures indicated a good parsimony and validity of our hypothesized model.

### Hypothesis testing

**Fig. 2:**
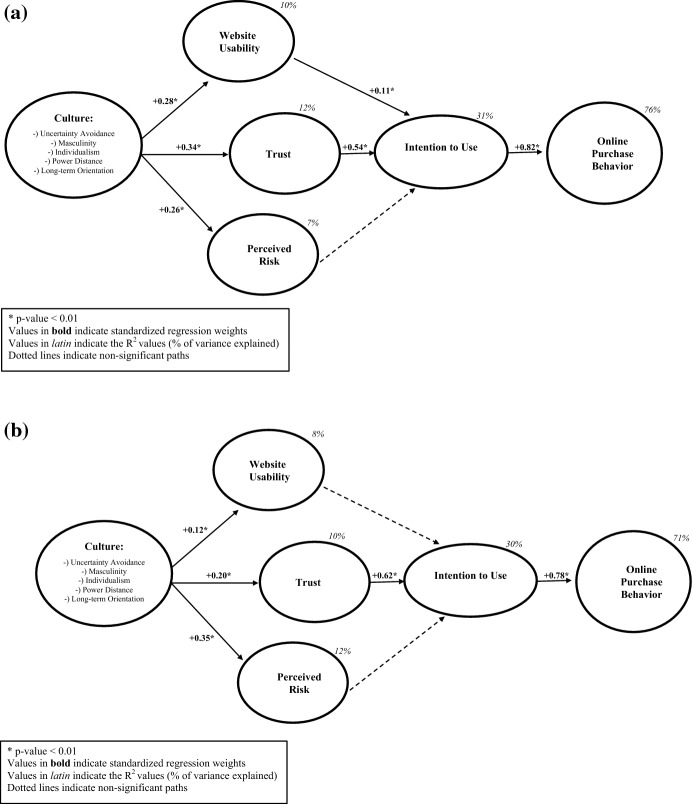
**a** Results of the structural model (European sample). **b** Results of the structural model (Asian sample)

We tested the hypothesized relationships using structural equation modeling (SEM) with AMOS (v.25; Arbuckle, 2013). Because our aim was to simultaneously test all the statistical influences among the variables, SEM represented one of the most appropriate statistical methods (Bagozzi & Yi, 1988; Hair et al., 2006). Our proposed structural models showed acceptable fit indexes: for Europe, χ^2^/df = 2.055, GFI = 0.970, RMSEA = 0.052, CFI = 965; for Asia, χ^2^/df = 1.965, GFI = 0.982, RMSEA = 0.048, CFI = 980. Hence, all the required values of goodness-of-fit indicators were respected (Bagozzi & Yi, 1988; Bentler, 1990). Figure 2a, b illustrate the results of the structural models, indicating both the standardized regression weights (the statistical influences among variables) and the *R*^2^ values (% of variance explained by the variables).

In both the European (Fig. 2a) and Asian (Fig. 2b) samples, *culture* significantly influenced *website usability* (β =  + 0.28, *p* < 0.01; β =  + 0.12, *p* < 0.01, respectively), *trust* (β =  + 0.34, *p* < 0.01; β =  + 0.20, *p* < 0.01, respectively), and *perceived risk* (β =  + 0.26, *p* < 0.01; β =  + 0.35, *p* < 0.01, respectively). Hence, *H1a*, *H1b, H2a, H2b*, *H3a,* and *H3b* are statistically supported. Interestingly, while the European cultural dimension mostly influenced consumer trust (β =  + 0.34, *p* < 0.01), the Asian cultural dimension mostly influenced consumer perceived risk (β =  + 0.35, *p* < 0.01).

In the European sample, *website usability* significantly influenced *intention to use* (β =  + 0.11, *p* < 0.01), thus supporting *H4a*. Similarly, *trust* had a significant effect on *intention to use* (β =  + 0.54, *p* < 0.01), thus providing statistical support for *H5a*. Perceived risk was not a significant predictor of intention to use (*p* > 0.05), thus H6a was not confirmed. The model was able to explain about one-third of the variance of intention to use (*R*^2^ = 31%). Finally, intention to use significantly and highly influenced online purchase behavior (β =  + 0.82, *p* < 0.01), thus supporting *H7a*. Overall, the proposed model was able to explain roughly two-thirds of the variance of online purchase behavior (*R*^2^ = 76%).

In the Asian sample, *intention to use* was significantly and highly predicted only by *trust* (*β* =  + 0.62, *p* < 0.01) but not by *website usability* (*p* > 0.05) nor *perceived risk* (*p* > 0.05), thus providing statistical support for *H5b*, but not *H4b* nor *H6b*. In this case, the model explained almost one-third of the variance of *intention to use* (*R*^2^ = 30%). Finally, *intention to use* was a strong predictor of online purchase behavior (*β* =  + 0.78, *p* < 0.01), thus supporting *H7b*. Overall, the model explained almost two-thirds of the variance of online purchase behavior (*R*^2^ = 71%).

## Discussion

The results that emerge from the analysis highlight a difference in the online purchasing process due in part to the different cultural values of European compared to Asian consumers. This is particularly true when considering *trust* and *website usability* which are influenced by culture in a stronger way in Europe compared to Asia (Baptista & Oliveira, 2015; Chen & Dibb, 2010). The reasons for this are explained by Hofstede’s (2001) cultural sub-dimensions of *masculinity* and *individualism*. European societies are typically masculine since they are based on values ​​such as assertiveness, ambition, power, materialism, and competitiveness; and they are also individualistic, or determined to achieve personal and not collective goals. We have previously validated with the reference literature that a high degree of *masculinity* implies a perception of higher user-friendliness of the platform. Moreover, a high degree of *individualism*, as found in most European societies, greatly influences the effect of *trust* on the *intention to use* a website (Venkatesh & Morris, 2000). Culture influences *perceived risk* more in Asia than in Europe (Hwang & Lee, 2012; Yoon, 2009) since *uncertainty avoidance* and the *power distance* cultural sub-dimensions are stronger in Asian culture. *Power distance*, or the acceptance of a rigid hierarchical structure and disparity between people, has a higher value in Asian than in European countries and is a dimension necessarily linked to the *perception of risk,* as it reflects the role of authority or superiors in making decisions. Consumers who come from a country with a high level of power distance like those in Asia believe more strongly that they can be deceived by unethical behavior on the part of a salesman than do consumers with a low power distance like those in Europe (Grazzini et al., 2020).

Similar reasoning can be used for *uncertainty avoidance*, which Hofstede (2001) defined as the extent to which members of a culture feel threatened by ambiguous or unknown situations. The Asian higher *perception of risk* in shopping online is explained by this cultural value. Our results are a strong confirmation of the assumption that culture and its specific components have to be carefully considered in managerial contexts to define online strategies. Hence, the present research contributes to literature on online purchase behavior by observing the way cultural dimensions affect consumer perceptions of an e-commerce platform and their subsequent acceptance and usage behaviors. Specifically, we observed how masculinity/femininity, short/long-term orientation, individualism/collectivism, and low/high power distance have a simultaneous effect on website usability, trust and perceived risk, which affect the intention to use the e-commerce platform and online purchase behavior. Therefore, e-commerce platforms operating in international markets should design their websites addressing these differences.

Managerial implications can be derived from our research. First of all, we noted how *website usability* influences *intention to use* in Europe but not in Asia. This means that e-tailers should invest more on this variable to successfully target the European market. European consumers are highly affected by the design of the website, including attractiveness, the ease of finding information, and the graphic aspect in general; therefore, the colors, the product images, and the type of font used are important. European users search for a uniform and easy navigational scheme that can help them access the various sections of the site.

A similar conclusion is reached about the influence of *trust* on the *online purchase behavior* variable, which is significant only for European consumers. Company policies regarding assistance and customer service need to be revised in order to increase consumer trust in Europe. Shipments and order tracking could be improved. Taking the Aliexpress platform as an example, deliveries from China offer a free shipping service and the sellers ship the goods with the cheapest and slowest providers. This implies that delivery time is a critical factor as a possible delay in delivery or a loss of an ordered product can generate a lot of distrust in the consumer towards the platform, since insecurity and the probability of suffering an economic loss prevail over the intention to purchase. Making traceability of all orders available regardless of the cost and shipping method can improve customer trust. The last consideration concerns the return policy in case of lack of conformity with the original sales conditions or product malfunction. This is a very controversial point because on Aliexpress, for example, consumers can receive a full or partial refund if the item does not match the description by contacting the seller directly (the Alibaba platforms act as intermediaries between seller and buyer), but the shipping fee is shouldered by the buyer. As a consequence, it is sometimes less costly to keep the product and agree directly with the seller on the amount of the refund.

Moreover, under the conditions of use of the service on marketplaces such as Alibaba.com, it is emphasized how the e-tailer takes no responsibility for the occurrence of any problems or disputes between a buyer and a vendor. It is up to the user to make sure that the supplier is reliable (for example, by considering only those companies whose identity is officially verified by the e-commerce platform). A further important consideration at the managerial level is that the *perceived risk* variable influences online purchasing behavior more in Asia than in Europe, even when there is little difference.

For both geographic markets, we can conclude that e-tailers must reduce the perception of risk by following two directions: payment methods; and sellers and quality controls. In terms of payment, safety conditions are strongly related to risk perception. For instance, the Alibaba platform accepts payments by credit cards from Mastercard and Visa, but a major guarantee on the protection of purchases is associated with Alipay, an online third-party payment platform launched in 2003 by Alibaba Group, which benefited from a first-mover advantage as it was the first electronic payment system introduced in China (Hu, 2020). Alipay allows payments without the need for credit cards or a bank account, but directly by paying with a mobile phone. Although the Alipay system is very widespread in Asia, in Europe it is still in its infancy.

Finally, in terms of quality, marketplaces such as Alibaba could, for example, implement more accurate control on sellers that use its platforms to avoid the occurrence of counterfeit product sales. What is missing is a rigid regulation on these platforms, and the probability of incurring in some scam is high. Therefore, clear and effective rules for consumer protection that provide a responsibility for sellers, brand protection, or consumer safety should be enacted.

## Limitations and future research

The research results confirm the different patterns among Asian and European consumers in their online purchasing behavior. Despite these important outcomes, it is still necessary to deal with this phenomenon by extending it or re-exploring the hypotheses. Our research is, in fact, limited in some respects.

The first limitation on the research concerns our sampling procedure: the sample is not probabilistic because it includes several nationalities, but does not include all nationalities in Asia and Europe, moreover we did not consider intra-group differences; therefore, the sample is not representative of the whole Asian or European continents. Furthermore, the cross-sectional nature of the study does not prevent a sample being homogeneous or composed of subjects belonging to the same social group. Some other limitations are linked to the methodology. Although the structural equation model is among the main methodologies used to analyze consumer behavior, more advanced techniques have recently been developed to test mediation using a bootstrap approach. This means that starting from the collected sample, many new samples are created of equal number randomly extracted with replacement, for example a participant can be extracted several times in a sample. Hayes' (2009) mediation and moderation modeling allows for repeated random sampling with substitution from the data set to calculate the desired statistics in each resampling, and all the bootstrap samples provide an approximation of the sampling distribution of the statistics of interest (see Zollo et al., 2017). In this respect, we suggest future scholars to expand our conceptual model (Fig. 1) by considering more mediating and moderating influences. For example, the effect of consumers’ perceived risk on website usability (Belanche et al., 2012) or the effect of website usability on trust (Chen & Dibb, 2010) might be investigated to enrich our hypothesized framework and provide more theoretical and practical implications. Further, our conceptual model might be better developed by adding all the Hofstede’s (2011) cultural sub-dimensions (i.e., including the *indulgence vs. restraint* dimension) or considering different and alternative categorizations of consumers’ cultural dimensions—see for example McSweeney’s (2002) criticism to Hofstede’s (2001) model.

Finally, we suggest future research to explore the same phenomenon by comparing more than one online platform as a term of comparison for Alibaba (for example, YOOX or ASOS) or to include more variables (for example, by integrating the sixth cultural sub-dimension of Hofstede’s (2011) model–*indulgence* vs. *restraint*, or outcome variables such as *word of mouth*) in the research. Moreover, respondents’ level of experience in online shopping might be included as a control variable of the study (see Hernández et al., 2010), thus assessing its impact on consumers’ purchase attitude and intention in e-commerce.
